# Clinical and Imaging Characteristics of Smear Negative Pulmonary Tuberculosis Patients: A Comparative Study

**DOI:** 10.1155/2024/2182088

**Published:** 2024-03-06

**Authors:** Alem Alemayehu, Liya Wassie, Sebsib Neway, Samuel Ayele, Abraham Assefa, Kidist Bobosha, Beyene Petros, Rawleigh Howe

**Affiliations:** ^1^College of Natural and Computational Sciences, Department of Microbial, Cellular and Molecular Biology, Addis Ababa University, P.O. Box 1176, Addis Ababa, Ethiopia; ^2^Armauer Hansen Research Institute (AHRI), P.O. Box 1005, Addis Ababa, Ethiopia; ^3^College of Health and Medical Sciences, School of Medical Laboratory Science, Haramaya University, P.O. Box 138, Dire Dawa, Ethiopia

## Abstract

**Background:**

Prevalence surveys in Ethiopia indicate smear negative pulmonary tuberculosis (SNPTB) taking the major share of the overall TB burden. It has also been a diagnostic dilemma worldwide leading to diagnostic delays and difficulty in monitoring treatment outcomes. This study determines and compares the clinical and imaging findings in SNPTB and smear positive PTB (SPPTB). *Methodology*. A case-control study was conducted on 313 PTB (173 SNPTB) patients. Data and sputum samples were collected from consented patients. Smear microscopy, GeneXpert, and culture analyses were performed on sputum samples. Data were analyzed using Stata version 17; a *P* value < 0.05 was considered statistically significant.

**Results:**

Of the 173 SNPTB patients, 42% were culture positive with discordances between test results reported by health facilities and Armauer Hansen Research Institute laboratory using concentrated smear microscopy. A previous history of TB and fewer cavitary lesions were significantly associated with SNPTB.

**Conclusions:**

Though overall clinical presentations of SNPTB patients resemble those seen in SPPTB patients, a prior history of TB was strongly associated with SNPTB. Subject to further investigations, the relatively higher discrepancies seen in TB diagnoses reflect the posed diagnostic challenges in SNPTB patients, as a higher proportion of these patients are also seen in Ethiopia.

## 1. Introduction

According to the World Health Organization (WHO), more than 40% of the 5.2 million pulmonary TB (PTB) patients were not bacteriologically confirmed but rather diagnosed based on signs and symptoms and chest imaging findings. Globally, only 33% of the 5.8 million newly diagnosed TB patients access the most sensitive molecular test particularly MTB/RIF assay (GeneXpert) as an initial diagnostic test. Moreover, SNPTB has been also a significant challenge in HIV-positive TB patient who requires earlier diagnosis and treatment [1]. Globally, Ethiopia has one of the highest proportions of smear negative pulmonary tuberculosis (SNPTB) among all patients with PTB. That may be attributed to the limited availability of reagents for routine TB laboratory diagnosis, lack of expertise, and poor adherence to WHO clinical algorithms for SNPTB diagnosis.

In Ethiopia, TB laboratory diagnosis mostly bases on direct acid-fast bacilli (AFB) smear microscopy, which is often insensitive and can only detect below 35% of TB patients [2]. Application of WHO-recommended rapid molecular tests is limited [1]. The rapid molecular test (GeneXpert) which was introduced in Ethiopia in 2012 detects only 63% of culture-positive SNPTB [3], and its full implementation has been hindered by different factors including limited cartridge supply and shelf life [4].

Clinical signs and symptoms and chest imaging findings remain the main PTB diagnostic tools for those PTB patients who are negative for sputum AFB or GeneXpert test [5, 6]. However, the nonspecificity of both clinical and chest imaging findings for PTB may often lead to incorrect diagnosis and result in inappropriate or delayed therapy [7–10]. Smear negative PTB remains less well studied, particularly in the current context in Ethiopia. In the present study, we evaluated clinical, radiological, and microbiological diagnostic information from SNPTB and SPPTB patients. Importantly, we compared microbiological assessment from health center (HC) laboratories where patients were initially diagnosed with analogous data obtained from the AHRI TB laboratory (AHRIL).

## 2. Methods

### 2.1. Study Setting

This is an unmatched case-control study conducted between August 2018 and August 2021. According to the national TB programmatic guideline (Figure S1) [11], a total of 313 newly diagnosed, adult (age ≥ 18 years), PTB patients who attended TB clinics were enrolled in the study from selected HC in Addis Ababa, Ethiopia. Patients who were initiated on anti-TB treatment for more than 5 days prior to enrollment, those who relapsed or were on retreatment, or those who were unable to provide sputum samples at the time of enrollment were excluded. Of the 313 PTB patients, 181 (58%) were diagnosed in public health facilities, 117 (37%) were from private health facilities, and the remaining 15 (5%) were identified through the community TB outreach system.

To keep test accuracy and patient classification uniform, concentrated smear acid-fast staining was performed at Armauer Hansen Research Institute's (AHRI) laboratory on all sputum samples, where a previous study on such method shown to enhance AFB detection by over 10% [12]. Cases (SNPTB patients) and controls (SPPTB patients) were defined based on the concentrated AFB (cAFB) findings of the AHRI laboratory (AHRIL). Briefly, SNPTB patients were defined as patients who have been clinically diagnosed as SNPTB at health institutions based on the Ethiopian TB diagnostic algorithm [11] and enrolled for anti-TB treatment and later were confirmed to be AFB negative at AHRIL. Similarly, SPPTB patients were defined as patients diagnosed as SPPTB at health institutions based on the Ethiopian TB diagnostic algorithm [11] and enrolled for anti-TB treatment and were also confirmed to be AFB positive at AHRIL.

Sociodemographic and clinical data were collected using structured questionnaires either as binary or continuous variables. Imaging findings were also recorded from patients' chest radiography report sent from different health facilities. Imaging reports with a specific presentation were included and copied to a prespecified classification format on the questionnaire [13].

### 2.2. Sample Collection and Processing

Five to ten milliliters of sputum were collected from each patient and transported to the AHRIL using a cold box. Upon arrival, samples were decontaminated using N-acetyl L-cysteine-sodium hydroxide (NALC-NaOH) and subjected to AFS, GeneXpert, and Lowenstein-Jensen (LJ) culture, and any leftover samples were refrigerated until further use. GeneXpert was performed for 109 consecutive samples according to the WHO implementation manual [14]. All mycobacteriology laboratory safety procedures were followed as described in the global laboratory initiative biosafety manual [15] and internal standard operating procedures.

### 2.3. M. tuberculosis Identification Using Lowenstein-Jensen Culture Growth Media

LJ culture analyses were performed following previously reported protocol [16]. Briefly, the NALC-NaOH-treated sputum samples were centrifuged, and the sediment was inoculated onto an egg-based LJ culture medium. Two LJ medium tubes, with 0.6 glycerol, were prepared for every sputum sample inoculation. All inoculated tubes were incubated at 37°C and growth of *M. tuberculosis* complex (MTBC) was checked weekly for a month. Typical MTBC colony morphology and subsequent AFS and Capilia neo-TB test (TANUS Laboratories, Japan) were used to confirm bacterial growth [17].

All data were captured onto SPSS version 25 (IBM, USA) and exported to Stata version 17 for analysis. Crude odds ratio (COR) and adjusted odds ratio (AOR) were determined for association between the study variables and PTB classification. Variables with *P* value < 0.2 in COR analysis and the patient's HIV status were included in the multivariable logistic regression analysis (MVA). Sensitivity and specificity with a 95% confidence interval of smear microscopy and GeneXpert test were also performed. In all analyses, a *P* value < 0.05 was considered statistically significant. The study was approved by the Institutional Research Ethics Review Board of the College of Natural and Computational Sciences, Addis Ababa University (IRB/030/2017) and the AHRI ethical review committee (PO44/17). All study participants provided written informed consent before enrollment in the study.

## 3. Results

A total of 173 and 140 participants were categorized as SNPTB and SPPTB patients, respectively, at AHRIL. Sixty-six of the SNPTB patients who were diagnosed as AFB negative at the health facilities were later diagnosed as AFB positive at AHRIL and were regrouped into the control. On the other hand, 33 patients (23 GeneXpert positive) who were diagnosed with SPPTB at the facilities were found to be negative following concentrated smear microscopy at AHRIL and were regrouped under the SNPTB (Table 1).

### 3.1. TB-Related Clinical Presentations among Smear Negative and Positive Patients

The clinical, imaging, and sociodemographic characteristics of the study population are summarized in Table 2. Almost all TB-related symptoms were present in both SNPTB and SPPTB patients in a similar proportion, except for the absence of fever, previous TB disease, and normal body mass index (BMI), which were more common among SNPTB patients compared to SPPTB patients and the odds of being SNPTB among patients with previous TB was nearly three times higher than those in SPPTB patients (*P* value < 0.05) (Table 2). Further accurate analysis of the study variables for patients with a concordant classification at the health centers and AHRI also exhibited a similar result (Table S1).

### 3.2. Imaging Findings among Smear Negative and Positive Patients

A total of 175 chest imaging findings were collected from all participants, and the proportion of patients with cavitary lesion was significantly lower among SNPTB (18%, *N* = 104) compared to SPPTB (39%, *N* = 71) (*P* < 0.05) (Table 3). Imaging findings excluding cavitary lesions, pleural effusions, and infiltration/consolidation were three times higher among SNPTB than SPPTB patients (*P* < 0.05) (Table 3).

Multivariate analysis of selected variables showed a positive association of older age, absence of fever, and prior TB illness with SNPTB, whereas the presence of cavitary lesions was negatively associated with SNPTB (*P* value < 0.05) (Table 4).

### 3.3. Mycobacterial Laboratory Analysis of Smear Negative and Positive TB Patients

The proportion of LJ culture positivity among SNPTB was 42% (*n* = 173) and that of SPPTB was 82% (*n* = 140). GeneXpert was done for 109 patients (69 SNPTB and 40 SPPTB), and the overall sensitivity was 71.6%, where the specific sensitivities were 97% in SPPTB and 38% in SNPTB patients compared with LJ culture that was used as a gold standard (Table 5). GeneXpert MTB detection was increased by 20%, i.e., from 52% (*n* = 74) at HCs to 71.2% (*n* = 74) at AHRIL.

## 4. Discussion

Diagnosis of smear negative TB has been a challenge, contributing to treatment delay and inaccurate patient selection for anti-TB treatment. This study sought to evaluate the clinical, radiographic, and microbiologic characteristics of smear negative PTB in Ethiopia. A unique study design was applied, in which patients were recruited based on classifications defined in standard healthcare settings, who later were evaluated and regrouped based on the concentrated smear microscopy result performed at AHRIL.

The major findings are as follows: First, among the clinical and imaging findings of patients with concordant classification at HC and AHRI, we observed a statistically significant or borderline association between SNPTB and (a) increased prior history of TB, (b) decrease in cavitary lesions (or increase in other radiological findings), and (c) absence of fever; (d) however, there was no significant association between HIV and SNPTB. Secondly, there were significant inconsistencies in the diagnosis of SNPTB at HC and AHRIL. Although there was concordance between laboratory results in the majority of smear-positive and smear-negative patients, there were a surprising number of patients classified as smear-negative in HC and who were later identified as positive at AHRI lab, and vice versa. Furthermore, GeneXpert detected many patients who were found to be smear-negative; however, it still identified only about half of the SNPTB patients who were culture-positive. Also, many patients who started anti-TB treatment were observed to be negative for all microbial indices.

Evaluation of clinical findings revealed similarities in clinical presentation with some differences. SNPTB were more likely to have a prior history of TB, and this finding was consistent among culture-negative patients compared with culture positives. This has been noted in other studies [18, 19], although not commonly reported. The underlying reason for the association between SNPTB and previous TB is not clear though it was reported that patients with SNPTB had persistently activated TB-specific T cells after therapy [20], implying persistence of activating stimuli, which might conceivably predispose to future infections, yet, why this would be unique to SNPTB is not clear. Alternatively, it is possible that some of the SNPTB patients with prior history of TB had imaging findings misconstrued as active disease leading to misdiagnosis since, a clinical and radiological presentation similarity between patients with previous TB and had active TB and patients with previous TB and had no active TB [21]. Hence, further research will be required to determine if this finding is generalizable.

We also observed that SNPTB patients had significantly fewer cavitary lesions as it has been previously observed. Although the mechanism of cavitation in TB is still unclear, whether resulting from liquefication of caseation granulomas or following postobstructive tuberculous pneumonia, either scenario results in a large release of aerosolized bacilli both in other regions of the lung and its environment [22] resulting in a higher bacillary load in patients with cavitary lesions [23]. Why SNPTB has substantially reduced cavitary lesions implies either a distinct mechanism of pathogenesis or alternatively reflecting an earlier stage in the process where bacilli are less prominent.

Although SNPTB has been considered the peculiar characteristic of HIV-infected individuals as it was associated with sputum smear negativity by different reports [24], this was not the case in our study population, where the proportion of HIV positivity was fairly similar in both groups. Another study has also reported a similar observation [25]. The earlier study reports that increased SNPTB in HIV may be related to a reduced cavitary disease which depends on immunocompetence [26]. Contrarily, the policy of antiretroviral therapy initiation at high CD4+ counts and that our HIV-positive participants' average is not as immunocompromised as early studies, our study ended up without a statistically significant difference between PTB classification and patients' HIV status. Consistent with this possibility, we did not see a significant difference in cavitary disease in SNPTB and SPPTB in HIV patients.

Our finding that a significant number of PTB patients classified as smear negative TB were found to be smear positive at AHRIL with concentrated smear microscopy method partly related to differences in individual laboratory procedures affecting sensitivity and specificity. However, the sputum concentration procedure typically enhances about 10% [3] alone, which apparently does not account for the entire discrepancies, implying that additional reasons should be at play.

Currently in Addis Ababa, AFB tests on smears are being phased out and replaced by GeneXpert for initial diagnosis of TB. Although GeneXpert testing is free, in government clinics, it is not available in all diagnostic laboratories, and Ministry of Health diagnostic algorithms refer to the lack of availability of GeneXpert testing, hence implying AFB testing as an acceptable alternative. As a result, it is common for patients to visit multiple clinics, both public and private, thus complex continuity of care clouding diagnostic classifications. On top of this, it is possible that some clinicians confidently gave the diagnosis of TB without a smear test being done.

Although GeneXpert has been recommended because of its enhanced sensitivity for TB diagnosis, some studies have shown that it fails to detect a significant percentage of culture-proven TB [27, 28]. Our findings in this study clearly confirm this, showing that only 38% of culture-positive TB among SNPTB patients were GeneXpert-positive. This percentage is lower than other reports in Ethiopia that may reflect differences in lab approaches. Further studies should be done to examine in detail the actual practices of health clinics versus the recommended guidelines and probe reasons for divergence.

Our finding that a significant number of patients were diagnosed as SNPTB based on clinical and radiological findings only and were negative for all microbiological tests has also been observed in previous studies [29, 30]. In some cases, this may reflect the simple fact that even culture is not perfectly sensitive and may in part relate to ambiguities of obtaining consistent sputum samples. On the other hand, more comprehensive samples like bronchoalveolar lavage (BAL) have been shown to detect over 80% of clinically diagnosed smears and GeneXpert-negative TB [31]. This reveals that part of the problem may be in obtaining an appropriate bacillus-containing specimen by routine sputum sampling method.

On the other hand, it is also a matter of concern that SNPTB is overdiagnosed. For instance, community-acquired pneumonia (CAP) elicited by bacteria not responsive to commonly used empiric antibiotics [32] is responsive to antibiotics used in conventional or MDR TB therapy and may be misdiagnosed as SNPTB which may be wrongly construed as SNPTB. Also, atypical bacteria, such as *Chlamydophila pneumoniae*, well studied in Western settings, characteristically treated with fluoroquinolones or macrolide antibiotics [33], are less responsive to commonly used empiric therapy in Ethiopia but potentially responsive to TB drugs like rifampicin [34] may imply for SNPTB. Such bacteria, as well as other standard pneumonia bacteria resistant to currently used empiric therapy but responsive to TB therapy, may result in misdiagnosis of SNPTB leading to unnecessarily prolonged therapy. Therefore, the exact answer needs further follow-up studies as well as investigations on other possible etiologies with more sensitive samples including BAL and induced sputum.

The absence of data from patient physical examination that will be similarly important as clinical symptoms for SNPTB diagnosis and interpersonal observer's variation in interpreting imaging findings from various imaging technologies could be the limitations of this study. In addition, the classification of patients based on cAFB and inclusion of patients starting anti-TB drug treatment may decrease the number of patients with AFB-negative and culture-positive results. Though PTB patients larger than the calculated sample size were involved, the difference in the number of SNPTB (cases) and SPPTB (controls) may result in a false absence of statistical association in some of the variables.

## 5. Conclusions

SNPTB patients were more likely to have a history of prior TB, noncavitary lesions, normal BMI, and older age compared with SPPTB. We observed a significant discrepancy between the diagnosis of SNPTB in health facility settings and AHRI with concentrated smear microscopy. SNPTB remains a challenging diagnosis, and we recommend further operational research on SNPTB diagnosis.

## Figures and Tables

**Table 1:** Patients' classification based on concentrated smear microscopy (from AHRIL) and clinical diagnosis (from health centers), Addis Ababa, Ethiopia, 2021.

Classification of patients based on diagnoses at health centers		Classification of patients based on AHRIL's AFB result					
		SPPTB (controls)		SNPTB (cases)		Total	
		Freq	%	Freq	%	Freq	%
TB diagnosis confirmed by							
SPPTB	AFS	23	29.9	3	9.1	26	23.6
	GeneXpert	47	61.0	21	63.6	68	61.8
	Not indicated	7	9.1	9	27.3	16	12.6
	Total	77	70.0	33	30.0	110	100

SNPTB	^∗^Clinical and imaging findings	61	96.7	132	94.3	193	95.1
	Not indicated	2	3.2	8	5.7	10	4.9
	Total	63	31.0	140	69.0	203	100

SNPTB: smear negative pulmonary tuberculosis; SPPTB: smear positive pulmonary tuberculosis; AHRIL: Armauer Hansen Research Institute's laboratory. ^∗^Patients with negative laboratory result either by AFS, GeneXpert, or classified as SNPTB without any laboratory diagnosis at the health centers.

**Table 2:** Univariate analysis of study variables among SNPTB and SPPTB patients, Addis Ababa, Ethiopia, 2021.

Study variables		Health centers' classification				AHRIL's classification			
		SPPTB	SNPTB	SPPTB = 0SNPTB = 1		SPPTB	SNPTB	SPPTB = 0SNPTB = 1	
		Freq (%)	Freq (%)	COR	*P* value	Freq (%)	Freq. (%)	COR	*P* value
Gender	Female	49 (44.6)	89 (43.8)	0.97	0.905	55 (39.3)	83 (48)	1.43	0.124
	Male	61 (55.5)	114 (56)	1		85 (60.7)	90 (52)	11	

Age group	≥45	18 (16.4)	51 (25.1)	1.71	0.076	22 (15.7)	47 (27.2)	2	0.016
	<45	92 (83.6)	152 (75)	1		118 (84.3)	126 (73)	11	

Occupation	DL	70 (68.6)	146 (75)	0.8	0.409	89 (66.4)	127 (77)	0.57	0.032
	Non-DL	32 (31.4)	50 (26)	1		45 (33.6)	37 (22.6)	11	

Cough	No	0	8 (4)	1		2 (1.5)	6 (3.5)	2.47	0.273
	Yes	106 (96)	193 (95)	1		135 (98)	164 (96)	11	

Weight loss	No	15 (14.6)	42 (21.1)	1.57	0.171	23 (17)	34 (20.4)	1.24	0.464
	Yes	88 (85.4)	157 (79)	1		112 (83)	133 (80)	1	

Fever	No	27 (25.2)	62 (31.2)	1.37	0.239	35 (25.6)	54 (32)	1.45	0.139
	Yes	80 (74.8)	137 (69)	1		102 (75)	115 (68)	1	

Night sweeting	No	23 (21.5)	41 (20.8)	1		28 (20.6)	36 (21.4)	1	
	Yes	84 (78.5)	156 (79)	1.04	0.889	108 (79)	132 (79)	0.95	0.858

Chest pain	No	40 (38.5)	63 (31.7)	0.74	0.236	48 (35.8)	55 (32.5)	0.86	0.55
	Yes	64 (61.5)	136 (68)	1		86 (64.2)	114 (68)	1	

Shortness of breath	No	38 (13)	59 (20.1)	0.72	0.201	47 (36.7)	50 (30.3)	0.75	0.248
	Yes	62 (62)	134 (69)	1		81 (63.3)	115 (70)	1	

Hemoptysis	No	87 (87)	155 (82)	1.51	0.24	103 (80)	139 (86)	0.63	0.142
	Yes	13 (13)	35 (18.4)	1		26 (20.2)	22 (13.7)	1	

Previous TB	No	101 (92)	171 (84.2)	1		130 (93)	142 (82)	1	
	Yes	9 (8.2)	32 (15.8)	2.1	0.062	10 (7.1)	31 (17.9)	2.84	0.007

HIV serostatus	Positive	17 (17.7)	32 (19.2)	1.14	0.673	19 (15.8)	30 (21)	1.29	0.39
	Negative	79 (82.3)	135 (81)	1		101 (84)	113 (79)	1	

Underweight BMI	No	119 (69)	84 (60)	1.3	0.282	67 (60.9)	136 (67)	1.47	0.106
	Yes	54 (31.2)	56 (40)	1		43 (39.1)	67 (33)	1	

BCG scar	No	56 (61.5)	120 (67)	1.29	0.338	78 (66.7)	98 (64.5)	0.91	0.708
	Yes	35 (38.5)	58 (32.6)	1		39 (33.3)	54 (35.5)	1	

Alcohol use	No	25 (26)	43 (23.9)	1		36 (29.3)	32 (20.9)	1	
	Yes	71 (74)	137 (76)	0.89	0.693	87 (70.7)	121 (79)	0.64	0.111

SNPTB: smear negative pulmonary tuberculosis; SPPTB: smear positive pulmonary tuberculosis; COR: crude odds ratio (univariate analysis); BMI: body mass index; DL: daily labourer.

**Table 3:** Univariate analysis of imaging findings among SNPTB and SPPTB patients in Addis Ababa, Ethiopia, 2021.

Imaging outcomes		HC classification				AHRIL classification			
		SPPTB	SNPTB	SPPTB = 0 SNPTB = 1		SPPTB	SNPTB	SPPTB = 0 SNPTB = 1	
		Freq. (%)	Freq. (%)	COR	*P* value	Freq. (%)	Freq. (%)	COR	*P* value
Cavitary lesions	Yes	16 (47.1)	32 (21.9)	0.32	0.004	28 (39.4)	20 (18.3)	0.34	0.002
	No	18 (52.9)	109 (73)	1		43 (60.6)	84 (81.7)	1	

Infiltration/consolidation	Yes	13 (44.8)	43 (59.4)	0.62	0.257	24 (39.3)	32 (33.3)	0.77	0.444
	No	16 (55.2)	85 (66.4)	1		37 (60.7)	64 (66.7)	1	

Pleural effusion	Yes	1 (3)	24 (17.0)	6.77	0.066	9 (12.7)	16 (15.4)	1.25	0.616
	No	33 (76.1)	117 (83)	1		62 (87.3)	88 (84.6)	1	

Other^∗^	Yes	6 (22.7)	44 (44)	2.67	0.073	10 (21.3)	39 (52)	4.01	0.001
	No	16 (77.3)	56 (56)	1		37 (78.7)	36 (48.5)		

SNPTB: smear negative pulmonary tuberculosis; SPPTB: smear positive pulmonary tuberculosis. ^∗^Imaging findings other than those listed in the table (includes discrete fibrosis, nodules (discrete, poorly defined), hilar lymphadenopathy, nonspecific signs of pneumonia, and abdominal ascites).

**Table 4:** Multivariate analysis of factors associated with SNPTB, Addis Ababa, Ethiopia, 2021.

Study variables	SNPTB = 1; SPPTB = 0											
	Health center's classification				AHRIL's (cAFB) classification				Concordant by both classification			
	AOR	(95% CI)		*P* value	AOR	(95% CI)		*P* value	AOR	(95% CI)		*P* value
Female gender	0.81	0.35	1.88	0.627	1.25	0.61	2.57	0.542	1.7	0.5	5.76	0.394
Age ≥ 45 years	1.93	0.6	6.24	0.272	2.7	1.04	7.04	0.042	9.59	1.05	87.38	0.045
Nondaily laborer	_	_	_	_	0.63	0.28	1.41	0.263	2.09	0.54	8.04	0.284
No weight loss	0.74	0.28	1.98	0.548	_	_	_	_	_	_	_	_
No shortness of breath	0.49	0.21	1.18	0.112	_	_	_	_	0.68	0.19	2.39	0.549
No fever	_	_	_	_	1.96	0.88	4.39	0.102	4.88	1.16	20.54	0.031
No hemoptysis	_	_	_	_	0.3	0.12	0.76	0.011	_	_	_	_
HIV positive	1.42	0.45	4.49	0.552	2.32	0.87	6.16	0.092	2.02	0.46	8.85	0.349
Previous TB	1.64	0.42	6.51	0.479	3.77	1.2	11.9	0.023	8.62	0.92	81.02	0.06
Normal BMI (≥18.5)	_	_	_	_	1.76	0.82	3.77	0.144	3.61	1.01	12.85	0.048
Cavitary lesions	0.35	0.13	0.92	0.034	0.33	0.14	0.77	0.011	0.26	0.08	0.86	0.027
Pleural effusion	1.38	0.45	4.19	0.573								
Other imaging findings^∗^	_	_	_	_	2.78	0.62	12.56	0.184	1.03	0.17	6.25	0.978

SNPTB: smear negative pulmonary tuberculosis; SPPTB: smear positive pulmonary tuberculosis; cAFB: concentrated AFB. ^∗^Imaging findings other than those listed in the table (include discrete fibrosis, nodules (discrete, poorly defined), hilar lymphadenopathy, nonspecific signs of pneumonia, and abdominal ascites (only 1 patient)). _Variables that did not have significant *P* values in the univariate analysis.

**Table 5:** Diagnostic accuracy of AFS smear microscopy and GeneXpert MTB/RTF assay among SNPTB and SPPTB patients, Addis Ababa, Ethiopia, 2021.

Accuracy of AFS vs. GeneXpert compared to the gold standard detecting MTBC among PTB patients							
GeneXpert (pooled)	LJ culture (gold standard)						
	LJ culture positive	LJ culture negative	Total	AFS (pooled)	LJ culture positive	LJ culture negative	Total
Positive	30	19	49	Positive	38	2	40
Negative	12	48	60	Negative	29	40	69
Total	42	67	109	Total	67	42	109
Accuracy parameters							
	(95% conf. inter.)				(95% conf. inter.)			
Sensitivity	71.64%	63.18%	80.10%	Sensitivity	56.72%	47.41%	66.02%
Specificity	71.43%	62.95%	79.91%	Specificity	95.24%	91.24%	99.24%
PPV	80.00%	72.49%	87.51%	PPV	95.00%	90.91%	99.09%
NPV	61.22%	52.08%	70.37%	NPV	57.97%	48.70%	67.24%
Prevalence	61.47%	52.33%	70.60%	Prevalence	61.47%	52.33%	70.60%

Accuracy of GeneXpert detecting MTBC among SPPTB patients vs. among SNPTB patients							
GeneXpert	SPPTB				SNPTB		
	LJ culture positive	LJ culture negative	Total		LJ culture positive	LJ culture negative	Total
Positive	37	2	39	Positive	11	10	21
Negative	1	0	1	Negative	18	30	48
Total	38	2	40	Total	29	40	69
Accuracy parameters							
(95% conf. inter.)				(95% conf. inter.)			
Sensitivity	97.37%	92.41%	102.33%	Sensitivity	37.93%	26.48%	49.38%
Specificity	0.00%	0.00%	0.00%	Specificity	75.00%	64.78%	85.22%
PPV	94.87%	88.04%	101.71%	PPV			64.17%
NPV	0.00%	0.00%	0.00%	NPV	62.50%	51.08%	73.92%
Prevalence	95.00%	88.25%	101.75%	Prevalence	42.03%	30.38%	53.68%

SNPTB: smear negative pulmonary tuberculosis; SPPTB: smear positive pulmonary tuberculosis; PPV: positive predictive value; NPV: negative predictive value.

## Data Availability

Additional data used to support the findings of this study are available from the corresponding author upon request.

## Supplementary Materials

Supplementary 1 Figure S1. Diagnostic algorithm of presumptive pulmonary TB patients (adapted from the National TB Program Guideline, Ministry of Health, Ethiopia, 2017).

Supplementary 2 Table S1. Clinical and imaging findings of patients who had a concordant classification at Health Centers and AHRI, 2021, Addis Ababa, Ethiopia.
