# Supplementary material for: Clinical and Imaging Characteristics of Smear Negative Pulmonary Tuberculosis Patients: A Comparative Study
Source: Pulm Med. 2024 Mar 6;2024:2182088. doi: 10.1155/2024/2182088 (PMC10937078; doi:10.1155/2024/2182088)
Supplement: Supplementary 1 — Figure S1. Diagnostic algorithm of presumptive pulmonary TB patients (adapted from the National TB Program Guideline, Ministry of Health, Ethiopia, 2017). [file 2182088.f1.docx]

| Two AFB negative result]  ^1In seriously sick patients for whom “HIV test is not done”, available investigations including Xpert, CXR and HIV testing may be done in one -go to avoid delays and save patients’ lives as such patients are advised to be managed at Hospital level.^  ^2Liquid specimens from EPTB site may be subjected to Xpert test without additional processing.^  ^3Broad spectrum antimicrobials excluding fluroquinolone or anti-TB drugs is to be given for 10- 14 days^  ^4One RR-TB Xpert result in population groups with low DR-TB risk (<5%) needs to be repeated on fresh specimen, if repeat test detects RR-TB; link to TIC for second line Anti-TB; if repeat test only detects MTB but not RR-TB; initiate first-line Anti-TB treatment and monitor response.^  =>Consider alternative Dx and treat^r4^  =>if seriously sick, refer for Xpert, CXR, pathology & Consult expert for “Clinical TB Dx’’  =>P/Pos TB  =>Link to TB clinic for TB Rx  =>Do FL-DST at base line using Xpert or FL- LPA  -Repeat Xpert if test result is in error; OR if MTB is not detected: Consider alternative Dx & treat^3^-if seriously sick, consult expert for “clinical TB Dx”  Give two sputum samples on spot for AFB  B (30-60 minutes apart)  Give three sputum samples (two on spot for AFB microscopy and send one for Xpert testing   - Patients with documented HIV infection - Seriously Sick patients^1^ - Children’s 2-4yrs of age - Prior TB treatment history - Contact history with RR/MDRTB patients (presumed proven) - Patients from congregated settings or health facilities - Presumed extrapulmonary TB^2^   Give one sputum for Xpert  Access to same day Xpert service  One or more AFB positive  MTB detected  MTB not detected  RR TB detected  Presumptive TB case |
| --- |

*AFS: Acid Fast Staining, CXR: Chest X-Ray, Dx: Diagnosis, Fl- DST: Fist Line-Drug Sensitivity Testing, FL-LPA: First Line -Line Probe Assay, TIC: Treatment Initiation Center, Rif: Rifampicin, RR: Rifampicin Resistant*

=> Repeat Xpert and decide on final diagnosis; patients^4^

=> treat as RR-TB at TIC if RR-TB is detected in patients with prior TB Rx
