# Supplementary material for: Clinical and Imaging Characteristics of Smear Negative Pulmonary Tuberculosis Patients: A Comparative Study
Source: Pulm Med. 2024 Mar 6;2024:2182088. doi: 10.1155/2024/2182088 (PMC10937078; doi:10.1155/2024/2182088)
Supplement: Supplementary 2 — Table S1. Clinical and imaging findings of patients who had a concordant classification at Health Centers and AHRI, 2021, Addis Ababa, Ethiopia. [file 2182088.f2.docx]

Table S1. Clinical and imaging findings of patients who had a concordant classification both at Health Centers and AHRI, 2021, Addis Ababa, Ethiopia

| Study variables | | SPPTB | | SNPTB | | Total | | SNPTB=1 SPPTB = 0 | |
| --- | --- | --- | --- | --- | --- | --- | --- | --- | --- |
|  |  | Freq. | % | Freq. | % | Freq. | % | COR | P -value |
| Gender | Female | 31 | 40.26 | 65 | 46.43 | 96 | 44.24 | 1.29 | 0.382 |
|  | Male | 46 | 59.74 | 75 | 53.57 | 121 | 55.76 | 1 |  |
| Age≥45 | Yes | 11 | 14.29 | 40 | 28.57 | 51 | 23.5 | 2.4 | 0.02 |
|  | No | 66 | 85.71 | 100 | 71.43 | 166 | 76.5 | 1 |  |
| Daily laborer | No | 24 | 31.58 | 29 | 21.17 | 53 | 24.88 | 0.58 | 0.094 |
|  | Yes | 52 | 68.42 | 108 | 78.83 | 160 | 75.12 | 1 | `_ |
| Cough | No | 0 | 0 | 6 | 4.32 | 6 | 2.8 | 1 |  |
|  | yes | 75 | 100 | 133 | 95.68 | 208 | 97.2 | 1 |  |
| Weight loss | No | 13 | 17.81 | 32 | 23.36 | 45 | 21.43 | 1.41 | 0.352 |
|  | Yes | 60 | 82.19 | 105 | 76.64 | 165 | 78.57 | 1 |  |
| Fever | No | 19 | 25 | 49 | 35.51 | 68 | 31.78 | 1.65 | 0.116 |
|  | Yes | 57 | 75 | 89 | 64.49 | 146 | 68.22 | 1 |  |
| Night sweeting | Yes | 61 | 81.33 | 109 | 80.15 | 170 | 80.57 | 0.93 | 0.835 |
|  | No | 14 | 18.67 | 27 | 19.85 | 41 | 19.43 | 1 |  |
| Chest pain | No | 26 | 35.62 | 41 | 29.71 | 67 | 31.75 | 0.76 | 0.381 |
|  | Yes | 47 | 64.38 | 97 | 70.29 | 144 | 68.25 | 1 |  |
| Shortness of breath | No | 26 | 40.63 | 38 | 59.38 | 64 | 33.83 | 0.65 | 0.165 |
|  | Yes | 42 | 30.66 | 95 | 69.34 | 137 | 66.17 | 1 |  |
| Hemoptysis | No | 10 | 14.29 | 19 | 14.5 | 29 | 14.43 | 1.02 | 0.967 |
|  | Yes | 60 | 85.71 | 112 | 85.5 | 172 | 85.57 | 1 |  |
| Previous TB | Yes | 4 | 5.19 | 26 | 18.57 | 30 | 13.82 | 4.16 | 0.011 |
|  | No | 73 | 94.81 | 114 | 81.43 | 187 | 86.18 | 1 |  |
| HIV serostatus | positive | 13 | 16.88 | 29 | 20.71 | 42 | 19.35 | 1.29 | 0.495 |
|  | negative | 64 | 83.12 | 111 | 79.29 | 175 | 80.65 | 1 |  |
| Underweight BMI | No | 40 | 51.95 | 88 | 63.31 | 128 | 59.26 | 1.6 | 0.109 |
|  | Yes | 37 | 48.05 | 51 | 36.69 | 88 | 40.74 | 1 |  |
| BCG scar | No | 39 | 59.09 | 81 | 63.78 | 120 | 62.18 | 1.22 | 0.524 |
|  | Yes | 27 | 40.91 | 46 | 36.22 | 73 | 37.82 | 1 |  |
| Alcohol use | yes | 18 | 41.86 | 25 | 58.14 | 43 | 22.28 | 0.69 | 0.303 |
|  | No | 50 | 33.33 | 100 | 66.67 | 150 | 77.72 | 1 |  |
| Cavitary lesions | Yes | 13 | 54.2 | 17 | 24.6 | 88 | 74.58 | 7.09 | 0.000 |
|  | No | 11 | 45.8 | 77 | 75.4 | 35 | 33.33 | 1 |  |
| Infiltration/ consolidation | Yes | 8 | 42.1 | 27 | 31.4 | 35 | 33.33 | 0.63 | 0.373 |
|  | No | 11 | 57.9 | 59 | 68.6 | 70 | 66.67 | 1 |  |
| Pleural effusion | Yes | 0 | 0 | 15 | 16 | 15 | 12.71 | 1 | _ |
|  | No | 24 | 100 | 79 | 84,0 | 103 | 87.29 |  |  |
| Other imaging | Yes | 3 | 18.6 | 37 | 53.6 | 40 | 47.06 | 5.01 | 0.019 |
|  | No | 13 | 81.3 | 32 | 46.8 | 45 | 52.94 | 1 |  |

SNPTB : smear negative pulmonary tuberculosis: SPPTB: smear positive pulmonary tuberculosis, COR: crude odds ratio (univariate analysis), BMI: Body mass index, - variables with no enough sample size to compute the statstics.
